# Comparison of 2D and 3D calculation of left ventricular torsion as circumferential-longitudinal shear angle using cardiovascular magnetic resonance tagging

**DOI:** 10.1186/1532-429X-11-8

**Published:** 2009-04-20

**Authors:** Iris K Rüssel, Sandra R Tecelão, Joost PA Kuijer, Robert M Heethaar, J Tim Marcus

**Affiliations:** 1 Department of Physics and Medical Technology, VU University Medical Center, Amsterdam, the Netherlands; 2 Department of Cardiology, VU University Medical Center, Amsterdam, the Netherlands; 3 Institute of Biophysics and Biomedical Engineering, University of Lisbon, Lisbon, Portugal

## Abstract

**Purpose:**

To compare left ventricular (LV) torsion represented as the circumferential-longitudinal (CL) shear angle between 2D and 3D quantification, using cardiovascular magnetic resonance (CMR).

**Methods:**

CMR tagging was performed in six healthy volunteers. From this, LV torsion was calculated using a 2D and a 3D method. The cross-correlation between both methods was evaluated and comparisons were made using Bland-Altman analysis.

**Results:**

The cross-correlation between the curves was *r*^2 ^= 0.97 ± 0.02. No significant time-delay was observed between the curves. Bland-Altman analysis revealed a significant positive linear relationship between the difference and the average value of both analysis methods, with the 2D results showing larger values than the 3D. The difference between both methods can be explained by the definition of the 2D method.

**Conclusion:**

LV torsion represented as CL shear quantified by the 2D and 3D analysis methods are strongly related. Therefore, it is suggested to use the faster 2D method for torsion calculation.

## Background

Left ventricular (LV) torsion is a sensitive marker for both systolic and diastolic dysfunction [1-3], and is therefore a useful addition to other strain measures such as radial, circumferential and longitudinal strain. LV torsion can be assessed using several techniques like speckle tracking echocardiography [4,5] and cardiovascular magnetic resonance (CMR) myocardial tagging [6,7]. However, there is still a debate on how to describe and calculate torsion in an optimal way, as a gold standard is not yet available. A straightforward determination method for LV torsion will facilitate clinical use of this measure.

**Figure 1:**
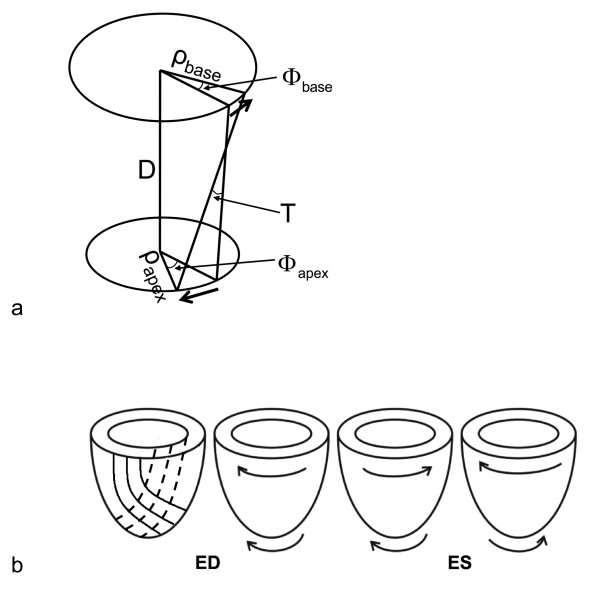
**(a) Torsion (T) defined as the CL shear angle (*ρ*: radius, *ϕ*: rotation, D: distance between slices)**. (b) Orientation of myofiber layers and normal rotational directions in the LV wall. Dashed lines: endocardial fiber direction, solid lines: epicardial fiber direction. ED: end-diastole, ES: end-systole.

Several methods to describe LV torsion have been previously published. First, the twist angle is used [8]. In this approach, the basal and apical rotations of the ventricle are subtracted, giving an indication of its twist. A second method is to divide this angle by the length of the ventricle, which describes a LV twist per unit length [9]. This parameter has the advantage of allowing the comparison of torsion between hearts of different sizes. A third method is to also take the radius of the heart into account. This describes torsion as the circumferential-longitudinal (CL) shear angle (Fig. 1) [10], which is completely comparable between hearts of different sizes and is directly related to fiber orientation and the processes in the cardiac wall during torsion (Fig. 1).

Calculating the CL shear angle in this way, is a fast approach that can be applied using only 2D short-axis (SA) image data. A method closer to the true CL shear angle, is the calculation of the local CL shear angle from extensive 3D strain analysis. However, this requires a dataset with SA and long-axis (LA) image data, from which 3D information on displacement of myocardium can be extracted. The additional acquisition time of the LA images and the large amount of additional post-processing time make 3D strain analysis time-consuming and less usable in clinical practice.

In this study, the results for CL shear strain from the 2D calculation and the 3D strain analysis methods are compared in healthy volunteers. The results will indicate whether it is sufficient to calculate torsion from only 2D SA CMR images.

## Methods

### Subjects

Six healthy male volunteers (26–56 years old, mean age: 43 years, ejection fraction: 56 ± 6%) with no history of cardiac disease were studied. Informed consent was obtained according to our institutional guidelines.

### Image acquisition

**Figure 2:**
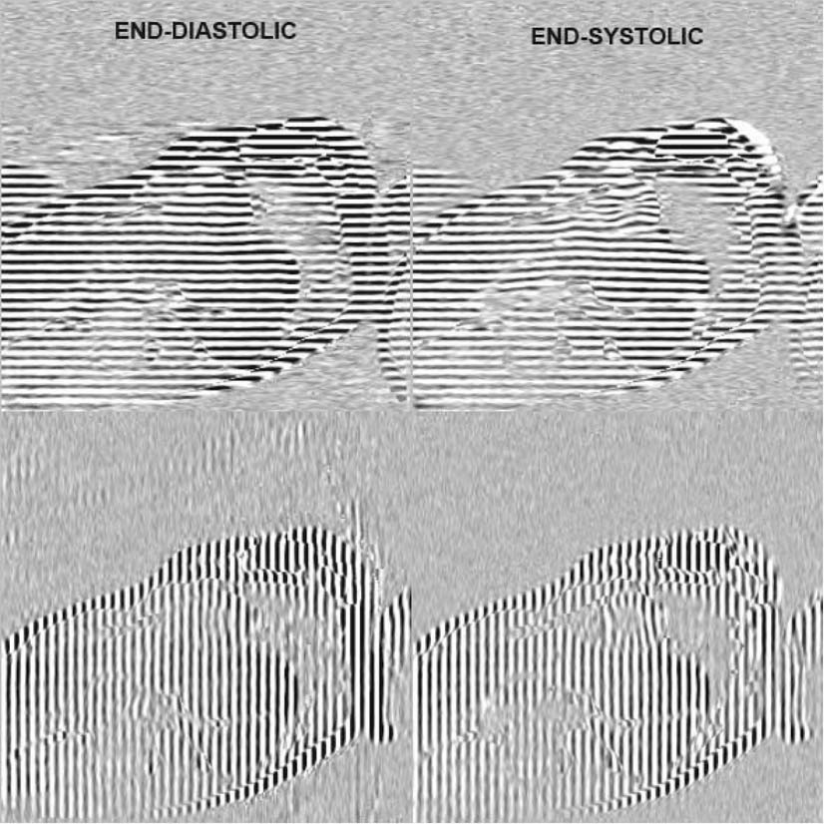
**Example of horizontally and vertically tagged CSPAMM images of an apical slice, showing rotation at end-diastole and end-systole**.

Imaging was performed on a 1.5 T whole body MR scanner (Magnetom Sonata, Siemens, Erlangen, Germany), using a four-element phased-array receiver coil. Cine imaging with complementary myocardial tagging (CSPAMM) was acquired with a steady state free precession (SSFP) sequence and a multiple brief expiration breath hold scheme as described in [11] (Fig. 2). Prospective triggering was used with a temporal resolution of 14 ms. The field of view (FOV) was 300 × 300 mm^2^, the excitation flip angle 20°, repetition time (TR) = 4.7 ms, echo time (TE) = 2.3 ms, receiver bandwidth (BW) = 369 Hz/pixel, imaging matrix size = 256 × 78. Five SA slices, evenly distributed over the LV, as seen on an end-systolic 4-chamber image, were acquired with both horizontal and vertical tagging. For the 3D analysis, three additional LA planes uniformly distributed around the LV and perpendicular to the SA with the tagging direction parallel to the SA slices were acquired. Tag line distance was equal to 7 mm.

### Post processing

Harmonic magnitude (HARM) and harmonic phase (HARP) images were computed from the SA and LA CSPAMM images as described in [12]. LV endocardial and epicardial contours were drawn on the HARM images using a dedicated software package (MASS, Medis, Leiden, the Netherlands). The myocardial tissue inside the contours was tracked by applying the previously described automatic extended HARP tracking method to the HARP images [13].

### Calculation of the CL shear angle using 2D analysis

For each tracked point in the basal, mid and apical SA slices, the rotation around the moving center of mass of the myocardium in the slice was calculated. Counterclockwise rotation as seen from apex to base was considered positive. Rotation (*Φ*) was averaged over the entire myocardium and set relative to the first timeframe. Two-dimensional torsion (*T*_2D_) between two slices can then be calculated as follows:

(1)

here shown for torsion between base and apex, where *D *is the distance between the slices and *ρ *the radius, calculated from the average pixel location inside the contours. In this way, the torsion can be interpreted as the global CL shear angle. The approach described in Eq. [1] has been previously applied by e.g. Aelen et al. and Delhaas et al. [10,14]. Another approach is to calculate the difference in circumferential displacement directly, which is geometrically closer to the 3D definition of shear angle (Fig. 1):

(2)

Analytically, the difference between Eq. [1] and Eq. [2] is:

(3)

generally resulting in larger values for *T*_2D_, since in the normal situation, (*ϕ*_apex _+ *ϕ*_base_) and (*ρ*_apex _- *ρ*_base_) will be positive.

Both definitions of torsion (Eqs. [1,2]) use the small angle approximation (tan *x *≈ *x*) for calculation of the shear angle and both will be used for comparison with 3D analysis. CL shear will be calculated at three levels: between base and apex, between base and mid, and between mid and apex.

### Calculation of the CL shear angle using 3D analysis

The longitudinal displacement of the LV was quantified by tracking the tag lines in the LA image planes [15]. The 3D displacement was obtained from interpolating the displacements between the LA planes and combining the trajectories on the SA planes with the trajectories on the LA planes [16]. A mesh of tetrahedrons was defined using the tracked points in intersecting regions of contours of neighbouring image planes [16]. The 3D Lagrangian strain tensor *E *was computed in RCL-coordinates with the knowledge of the displacements of the points, from which the CL shear angle (*α*_CL_) could be computed:

(4)

where *E*_ii _are diagonal elements and *E*_ij _are off-diagonal elements of the strain tensor, *ε *is the axial strain and i and j are indices of the circumferential and longitudinal direction, respectively. Therefore, three-dimensional torsion (*T*_3D_) is defined as: *T*_3D _= *α*_CL_.

The results were averaged over the entire 5 slices for base-apex torsion, the entire top 3 slices for base-mid torsion and the entire bottom 3 slices for mid-apex torsion to obtain a measure equivalent to that obtained with the 2D analysis.

### Comparisons and statistics

The 2D and 3D shear angle curves were compared to evaluate the difference between the two analysis methods. First, cross-correlations were calculated between the torsion curves obtained with both methods (base-apex, base-mid and mid-apex). The 2D and 3D curves were shifted with respect to each other. One time-lag was defined as the time between two subsequent cardiac phases (14 ms) as acquired during tagged CMR. Furthermore, paired T-tests and Bland-Altman analysis was performed on the global torsion curves. Limits of agreement for the Bland-Altman analysis were calculated using linear regression [17] in case of comparison with *T*_2D_.

Results are presented as mean ± SD. P-values below 0.05 are regarded as statistically significant.

## Results

### Cross-correlations

**Figure 3:**
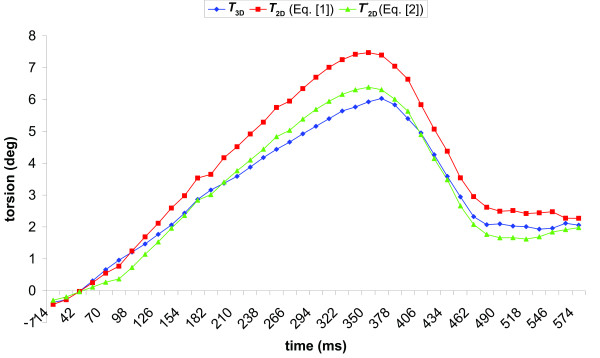
**Example of a global base-apex torsion curve from a healthy subject, calculated with the 2D (red: *T*_2D_, Eq**. [1]; green: T*_2D_, Eq. [2]**) and the 3D (blue: *T*_3D_, Eq**. [5]) **method**.

An example of torsion curves using the *T*_2D_, T*_2D _and *T*_3D _calculation methods is presented in Fig. 3. The average maximum cross-correlation over all regions (base-apex, base-mid and mid-apex) and subjects between the *T*_2D _and the *T*_3D _was high (r^2 ^= 0.97 ± 0.02, see Table 1) and there was no time delay between the curves. The highest correlation between the two calculation methods was obtained for the base-apex torsion (r^2 ^= 0.99 ± 0.01).

**Table 1:** r^2 ^values and time-delays for the cross-correlations between the 2D and the 3D torsion calculation method.

	**r^2 ^(*T*_2D_)**	r^2 ^(T*_2D_)	Time delay (time lags) (*T*_2D_, T*_2D_)
**Base-apex**	0.99 ± 0.01	0.98 ± 0.02	0.0 ± 0.0

**Base-mid**	0.97 ± 0.02	0.96 ± 0.03	0.0 ± 0.0

**Mid-apex**	0.95 ± 0.02	0.93 ± 0.06	0.0 ± 0.0

Average	0.97 ± 0.02	0.96 ± 0.04	0.0 ± 0.0

One time-lag corresponds to 14 ms. A negative delay would indicate that the 3D method was delayed with respect to the 2D method. Values are mean ± SD.

When using T*_2D_, the cross-correlation was slightly lower (r^2 ^= 0.96 ± 0.04, see Table 1), although the time delay between the curves was still zero.

### Comparison of 2D and 3D torsion

**Figure 4:**
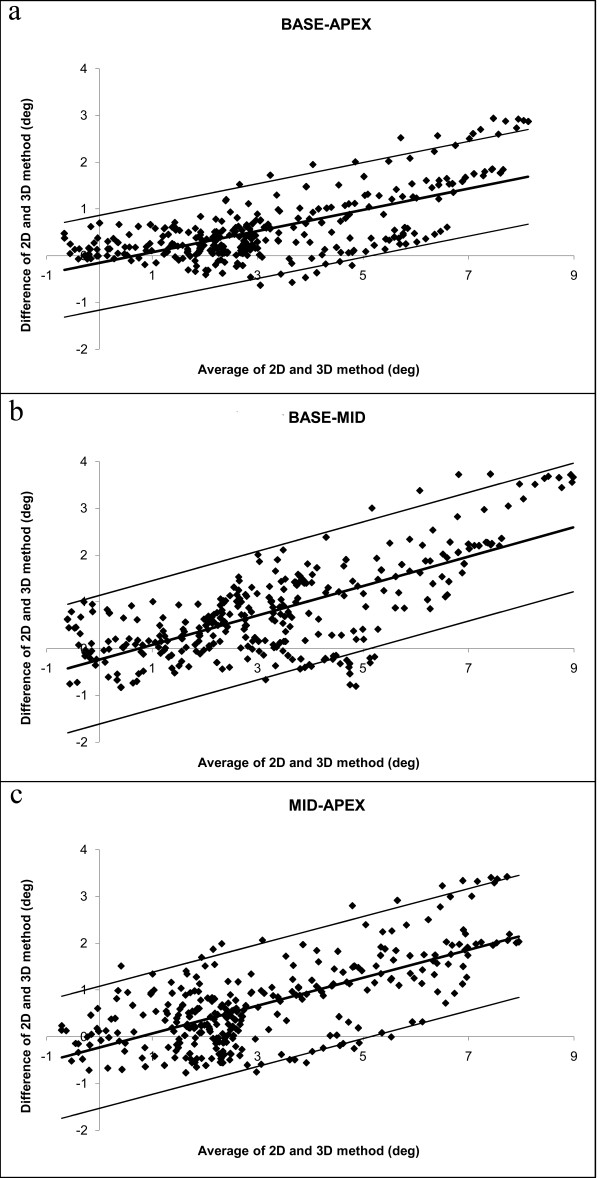
**Bland-Altman plots for the base-apex (a), base-mid (b) and mid-apex (c) torsion values of the subjects, using *T*_2D_**. The difference between the values from both methods increases linearly when the average torsion value becomes higher.

The torsion values obtained with *T*_2D _were significantly (p < 0.0001) higher than those obtained with *T*_3D _(Table 2). Bland-Altman analysis revealed a significant positive linear relationship between the difference in torsion and the average torsion (Fig. 4, Table 2). This linear relationship was observed in all curves (base-apex: r = 0.67; base-mid: r = 0.71; mid-apex: r = 0.69; all p < 0.0001). The limits of agreement are therefore calculated as a regression line [17] and can be found in Table 2.

**Table 2:** Comparison between the 2D and 3D torsion curves, top values: *T*_2D _as 2D method, bottom values: T*_2D _as 2D method.

	**Average values** **(3D vs. 2D) (deg)**	**Regression line** **(3D vs. 2D)**	**Correlation coefficient (Difference vs. Average)**	**Limits of agreement regression line (top) (Difference vs. Average), Limits of agreement (bottom) (Difference)**
**Base-apex**	2.9 ± 1.9 vs. 3.4 ± 2.3	y = 0.77*x + 0.21	r = 0.67	y = 0.23*x - 0.15 ± 1.01
	2.9 ± 1.9 vs. 2.9 ± 2.1	y = 0.86*x + 0.38	r = 0.39	0.03 ± 1.14

**Base-mid**	2.9 ± 1.9 vs. 3.7 ± 2.6	y = 0.70*x + 0.32	r = 0.71	y = 0.32*x - 0.23 ± 1.37
	2.9 ± 1.9 vs. 3.4 ± 2.4	y = 0.75*x + 0.32	r = 0.56	0.51 ± 1.71

**Mid-apex**	2.8 ± 1.8 vs. 3.5 ± 2.4	y = 0.71*x + 0.31	r = 0.69	y = 0.30*x - 0.23 ± 1.31
	2.8 ± 1.8 vs. 2.4 ± 2.0	y = 0.82*x + 0.81	r = 0.26	-0.39 ± 1.61

**Figure 5:**
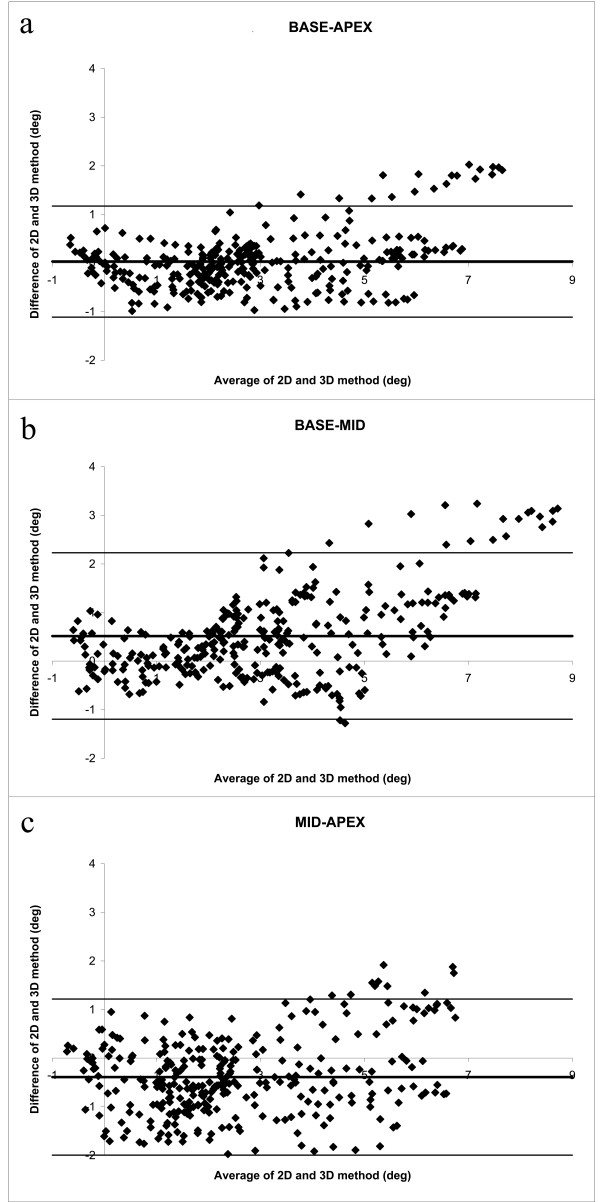
Bland-Altman plots for the base-apex (a), base-mid (b) and mid-apex (c) torsion values of the subjects, using T*_2D_. Notice that the linear relationship between difference and average is reduced as compared to Fig. 4.

When using T*_2D_, the difference between the 2D and 3D method between the base-apex curves was no longer significant (p = 0.35). For the base-mid curves, T*_2D _was significantly higher (p < 0.0001) and for the mid-apex curves T*_2D _was significantly lower (p < 0.0001). In the Bland-Altman analysis, only weak correlations were found between the difference and the average of both methods (Fig. 5, Table 2); therefore the limits of agreement were no longer calculated as a regression line. Limits of agreement are slightly increased using T*_2D_, however (Table 2).

## Discussion

### Comparison of 2D and 3D measurement methods

This study shows that there is a high correlation between global torsion calculated as the CL shear angle using the 2D and the 3D methods, and that the curves are not delayed with respect to each other. However, when the 2D CL shear is calculated as the LV twist per unit length, multiplied by the average radius (*T*_2D_, Eq. [1]), the difference between the 2D and 3D method increases as the torsion value increases. *T*_2D _gives the highest values. When the 2D CL shear is calculated as the difference in circumferential displacement per unit length (T*_2D_, Eq. [2]), the linear trend in the difference is much smaller.

Considering that *T*_3D _is obtained from information on local deformations following from 3D displacements, the *T*_3D _is expected to be closest to the true torsion.

A strong relationship and no time delay between curves obtained with the 2D and the 3D method was found.

For T*_2D_, no linear relationship between the difference and average of the 2D and 3D method was observed. However, only when torsion was calculated between the basal and apical plane, was there no bias between both methods. For the other two longitudinal regions (base-mid, mid-apex), respectively an overestimation and an underestimation of torsion were found with respect to *T*_3D_. For *T*_2D _on the other hand, the difference between the 2D and 3D methods was relatively constant over the longitudinal regions.

This might be explained by the difference between *T*_2D _and T*_2D_. Errors in the radius of the LV might be introduced by including too many trabeculae in the contours (underestimation of radius, usually on apical level), or by including pericardial fat in the contours (overestimation). Contours in tagged images are difficult to delineate, because of the low resolution of the HARM images. An overestimation of the radius in the basal slice or an underestimation of the radius in the apical slice will both lead to an overestimation of *T*_2D_, regarding the (*ρ*_base _- *ρ*_apex_) term from Eq. [3], causing the relatively constant overestimation of torsion over the longitudinal regions. From error-propagation (assuming similar variance in radii, and constant *D *and *Φ*) it can be derived that the variance in T*_2D _is larger than in *T*_2D_. Under- or overestimations of radii are directly reflected in the torsion value, but less so in T*_2D_, which also explains the slightly lower cross-correlation found with this method (Table 1).

Several other aspects might explain the differences between the 2D and 3D methods. Since the tetrahedrons in the 3D analysis can only be defined in the intersecting region of the contours of neighbouring image planes, the 3D analysis represents less myocardium than the 2D analysis [15]. The difference between the methods is probably not explained by the difference in the amount of myocardium used for averaging in the 2D and 3D methods, since the CL shear angle is thought to be constant over the transmural direction of the myocardial wall [8,18]. However, the difference might be an explanation for the somewhat reduced correlation at the apical level between the 2D and 3D methods (Table 1).

Since *T*_2D _is less sensitive to errors in the radius of the ventricle, its bias to *T*_3D _is relatively constant, and it has been often used in literature, this is probably the best method to calculate 2D CL shear. Furthermore, when reference data obtained with the same method is present, the observed deviation between *T*_2D _and *T*_3D _is less important.

### Clinical implications

Torsion calculated as the 2D CL shear angle is very fast, both in acquisition as in post-processing, as compared to the 3D analysis. Acquisition of a horizontally and vertically tagged CSPAMM CMR slice requires up to 5 minutes with the protocol that was used in this study. Post-processing for the 2D method is fully automatic, except for the contours that have to be drawn manually. The calculation part of the post-processing requires only a few minutes on a standard PC. The main drawbacks of the 3D analysis are that additional images have to be acquired, and that the accompanying contours need to be drawn. These extra LA images also require additional post-processing.

Both 2D methods show strong (cross-)correlation with *T*_3D_. The constant bias and narrower limits of agreement of *T*_2D_, together with the fact that *T*_2D _has already been used more often in literature, providing more reference data on torsion calculated in this way [8,10,19], demonstrates that this measure is suitable to be used in clinical practice.

### Limitations

In this study, no patients were included. It is known that torsion can be altered in several ways in patients with different diseases [2,20-24]. Therefore, comparisons should be made in patients with different alterations in torsion. This might be a topic of future investigation.

**Figure 6:**
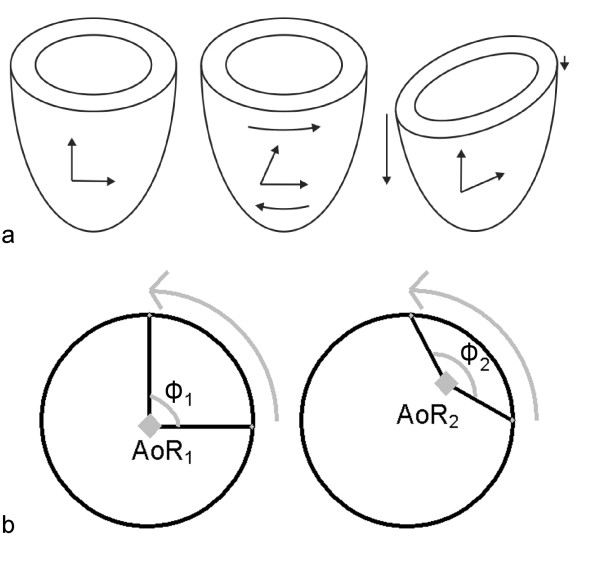
**(a) Two modes of deformation resulting in the same CL shear angle: left image: undeformed LV; middle image: LV deformed due to torsion; right image: LV deformed due to differences in longitudinal displacement**. (b) Influence of the location of the axis of rotation (AoR) on the observed rotation (*ϕ*) for the same displacement. The axis of rotation will be affected by conditions such as RV hypertrophy.

Furthermore, no comparisons were made for different (circumferential) regions in the LV. In the 3D method, differences in regional CL shear angle can be the result of two deformation modes: differences can be the result of either longitudinal displacement, or they can be due to torsion (Fig. 6) [25]. In the 2D method, differences observed in shear between circumferential segments are related to the choice of the axis of rotation for calculation (Fig. 6) [18,26,27]. Therefore, the origin of the unreliability in torsion calculated in circumferential segments between the 2D and 3D methods is different. Hence, it is expected that no strong relation between torsion in circumferential segments calculated by the 2D or the 3D method will be present.

## Conclusion

Global LV torsion represented as the CL shear angle quantified by a 2D method and a 3D method show a very strong relationship. Observed differences between both methods can be explained by the definition of the 2D method. Consequently, it is suggested to use the faster and easier 2D method for calculation of global LV torsion.

## Competing interests

The authors declare that they have no competing interests.

## Authors' contributions

IKR participated in study design, manuscript preparation, data analysis and interpretation. SRT participated in study design, data analysis and interpretation, manuscript revision. JPAK participated in study design, data interpretation and manuscript revision. RMH participated in study design and manuscript revision. JTM participated in study design, data collection and manuscript revision. All authors read and approved the final manuscript.
